# A randomized controlled trial of fresh frozen plasma for coagulopathy in Russell's viper (*Daboia russelii*) envenoming

**DOI:** 10.1111/jth.13628

**Published:** 2017-02-16

**Authors:** G. K. Isbister, S. Jayamanne, F. Mohamed, A. H. Dawson, K. Maduwage, I. Gawarammana, D. G. Lalloo, H. J. de Silva, F. E. Scorgie, L. F. Lincz, N. A. Buckley

**Affiliations:** ^1^ Clinical Toxicology Research GroupUniversity of NewcastleNewcastleNew South WalesAustralia; ^2^ South Asian Clinical Toxicology Research CollaborationFaculty of MedicineUniversity of PeradeniyaPeradeniyaSri Lanka; ^3^ Department of MedicineFaculty of MedicineUniversity of KelaniyaRagamaSri Lanka; ^4^ Department of PharmacologySOMSSydney Medical SchoolUniversity of SydneyNew South WalesAustralia; ^5^ Department of BiochemistryFaculty of MedicineUniversity of PeradeniyaPeradeniyaSri Lanka; ^6^ Department of MedicineFaculty of MedicineUniversity of PeradeniyaPeradeniyaSri Lanka; ^7^ Clinical Sciences and International Public HealthLiverpool School of Tropical MedicineLiverpoolUK; ^8^ Hunter Haematology Research GroupCalvary Mater NewcastleNewcastleNew South WalesAustralia

**Keywords:** antivenoms, consumption coagulopathy, plasma, snake venoms, snakebites

## Abstract

**Essentials**

- Russell's viper envenoming is a major health issue in South Asia and causes coagulopathy.
- We studied the effect of fresh frozen plasma and two antivenom doses on correcting coagulopathy.
- Fresh frozen plasma did not hasten recovery of coagulopathy.
- Low‐dose antivenom did not worsen coagulopathy.

**Summary:**

**Background**

Russell's viper (*Daboia russelii*) envenoming is a major health issue in South Asia and causes venom‐induced consumption coagulopathy (VICC).

**Objectives**

To investigate the effects of fresh frozen plasma (FFP) and two antivenom doses in correcting VICC.

**Methods**

We undertook an open‐label randomized controlled trial in patients with VICC at two Sri Lankan hospitals. Patients with suspected Russell's viper bites and coagulopathy were randomly allocated (1 : 1) to high‐dose antivenom (20 vials) or low‐dose antivenom (10 vials) plus 4 U of FFP. The primary outcome was the proportion of patients with an International Normalized Ratio (INR) of < 2 at 6 h after antivenom administration. Secondary outcomes included anaphylaxis, major hemorrhage, death, and clotting factor recovery.

**Results**

From 214 eligible patients, 141 were randomized: 71 to high‐dose antivenom, and 70 to low‐dose antivenom/FFP; five had no post‐antivenom blood tests. The groups were similar except for a delay of 1 h in antivenom administration for FFP patients. Six hours after antivenom administration, 23 of 69 (33%) patients allocated to high‐dose antivenom had an INR of < 2, as compared with 28 of 67 (42%) allocated to low‐dose antivenom/FFP (absolute difference 8%; 95% confidence interval − 8% to 25%). Fifteen patients allocated to FFP did not receive it. Severe anaphylaxis occurred equally frequently in each group. One patient given FFP developed transfusion‐related acute lung injury. Three deaths occurred in low‐dose antivenom/FFP patients, including one intracranial hemorrhage. There was no difference in recovery rates of INR or fibrinogen, but there was more rapid initial recovery of factor V and FX in FFP patients.

**Conclusion**

FFP after antivenom administration in patients with Russell's viper bites did not hasten recovery of coagulopathy. Low‐dose antivenom/FFP did not worsen VICC, suggesting that low‐dose antivenom is sufficient.

## Introduction

Snakebite continues to be a major health issue in the rural tropics, particularly in resource‐poor regions of Asia, Africa, and Latin America. Although it is difficult to estimate the numbers of envenoming cases and deaths each year, these are in excess of half a million and tens of thousands, respectively 1. The major clinical syndromes of snake envenoming are coagulopathy, neurotoxicity (paralysis), myotoxicity, local cytotoxicity/necrosis, and acute kidney injury. Venom‐induced consumption coagulopathy (VICC) is the most frequent serious complication, and can result in major hemorrhage and death 2, although the less common neurotoxicity carries a higher risk of a fatal outcome 3. Treatment includes antivenom and, potentially, clotting factor replacement, but the evidence to support their effectiveness in resolving VICC is limited; there are no placebo‐controlled trials of antivenom 4, and only one of fresh frozen plasma (FFP) 5. The majority of studies of FFP in VICC have been observational in nature 6, 7, 8, 9.

Russell's viper (*Dabaoi russelii*) is a snake of major medical importance in South and Southeast Asia 10, 11, 12. The major manifestation of envenoming is VICC, which is characterized by decreased levels of fibrinogen, factor V, FVIII, and FX, leading to a prolonged prothrombin time (PT: International Normalized Ratio [INR]) and a prolonged activated partial thromboplastin time (APTT) 13. The procoagulant activity of the venom is attributable to metalloproteinases, which are FX and FV activators 14. VICC is the most common indication for antivenom administration in patients with Russell's viper bites, although there is ongoing controversy over the dose of antivenom (initial dose of 10 or 20 vials), repeat antivenom dosing, and the use of factor replacement. We have recently shown that recovery of coagulopathy occurs over a period of 24–48 h in patients given 10 vials of antivenom 15. Even so, full recovery of clotting function takes up to 48 h, during which time the patient potentially remains at risk of bleeding. Theoretically, FFP administration should rapidly correct the coagulopathy in the absence of ongoing venom‐induced clotting factor consumption. This has been shown for Australian elapid snakebites, but has not been investigated in viper envenoming.

There is also concern that a low dose of antivenom is insufficient to neutralize all of the venom, and, followed by the administration of FFP, could re‐initiate the consumption process, extending the duration of the coagulopathy. In a study of FFP in Australian elapid envenoming causing VICC, the administration of FFP sped up the recovery of the coagulopathy, except when it was given < 6 h after the bite 5. However, the etiology of VICC is different for Russell's viper venom, which contains metalloproteinase FX and FV activators, rather than a serine protease prothrombin activator 16. The coagulopathy from the latter develops rapidly and appears to resolve irrespective of antivenom 17, whereas Russell's viper VICC appears to be slower in onset, and recovery is delayed unless antivenom is administered.

We hypothesized that FFP would hasten the recovery of the coagulopathy in patients with Russell's viper bite after the administration of 10 vials of antivenom. We compared this lower dose of antivenom plus FFP with high‐dose antivenom, to determine whether this resulted in more or less rapid correction of clotting function in patients bitten by Russell's vipers.

## Materials and methods

We undertook an open‐label randomized controlled trial comparing low‐dose antivenom (10 vials) plus FFP (4U) with high‐dose antivenom (20 vials) without FFP at two Sri Lankan hospitals in patients with VICC from Russell's viper envenoming. The study was approved by the Ethical Review Committee, University of Peradeniya, Sri Lanka, and the Human Ethics Research Committee of the University of Newcastle, Australia. All patients gave written and informed consent. The study was registered with the Sri Lankan Clinical Trials Registry (number SLCTR/2010/011).

### Study recruitment and patients

All patients (aged > 15 years) with a suspected snakebite presenting to the Base Hospital Polonnaruwa from October 2012 to March 2015, or the Base Hospital Kurunegala from May 2013 to March 2015, were recruited to a prospective cohort study. From all patients with suspected snakebites, we aimed to recruit patients with VICC to the randomized clinical trial. The inclusion criteria for the randomized clinical trial were a suspected Russell's viper (*D. russelii*) bite with coagulopathy defined as an abnormal 20‐min whole blood clotting test (20WBCT). Sufficient FFP also had to be available to administer within 4 h of commencement of antivenom administration. Exclusion criteria were a definite bite by a another snake, pregnancy, age < 16 years, allergy to blood products, and prior antivenom administration (such as at a primary hospital before transfer). Patients could also be excluded by their treating physician if they were not happy to give the doses of antivenom in the study, or wanted to determine whether FFP was to be administered.

### Treatment protocol

All patients with snakebites underwent an initial assessment by clinical research assistants, including collection of baseline clinical data, symptoms and signs of snake envenoming, 20WBCT, and information on snake identification. The study was explained to patients with a suspected Russell's viper bite and a positive 20WBCT by a clinical research assistant, in their native language (Sinhala, Tamil, or English). Written informed consent was then obtained, and the patients were randomized in a 1 : 1 ratio to either low‐dose antivenom (10 vials) plus FFP, or high‐dose antivenom (20 vials).

Block randomization was used for each of the two study hospitals to maintain balance at both sites. Blocks of 2 and 4 (e.g. AB, BA or AABB, ABAB, etc.) were randomly generated with Microsoft Excel, producing two separate random sequences of allocations. The random allocation was provided to clinical research assistants at each hospital from a central contact (F.M.), so that the patient, treating team, research assistants and investigators were all blinded to allocation. All patients received Indian polyvalent antivenom from VINS Bioproducts (Hyderabad, India) over a period of 1 h, with the low‐dose antivenom group receiving 10 vials and the high‐dose group receiving 20 vials. Those patients allocated to low‐dose antivenom and FFP then received 4 U of FFP (approximately 1000 mL; 10–15 mL kg^−1^) over a period of 30–60 min. The treating team cross‐matched and ordered FFP, and either the blood bank supplied FFP (daytime) or a senior house officer (night‐time) obtained FFP from the blood bank. Patients not allocated to FFP were not to receive FFP until after the 6‐h primary outcome. Any further antivenom administration was determined by the treating doctor(s), but was discouraged prior to the 6‐h primary outcome time.

All patients had a 10‐mL blood sample collected prior to antivenom administration (baseline), and then a further 5 mL collected at 1, 3, 6, 12 and 24 h after antivenom administration, and daily until discharge. Blood was collected in serum and citrated tubes, immediately centrifuged for 7 min at 3000 r.p.m., aliquoted, and frozen at − 20 °C for 1–2 weeks, before being transported in mobile freezer units to a central location to be stored at − 80 °C until the completion of the study. All samples were then sent to the Clinical Toxicology Research Group Laboratory, University of Newcastle, Australia for analysis. Thermocrons set to measure the temperature every 7 min were used to confirm that samples had not thawed.

### Data collection

Demographic information (sex and age), details of the bite, information on snake identification, clinical features of snake envenoming, 20WBCT results, treatment (trial treatment; repeat antivenom), adverse effects, complications (hemorrhage, acute kidney injury, and death) and study outcomes were recorded on clinical research datasheets, which were then entered into a relational database (Microsoft Access) by a single trained data entry research assistant. Baseline observations were recorded prior to antivenom administration. Blood pressure, heart rate, respiratory rate, oxygen saturation and any features of systemic hypersensitivity reactions were then recorded. Systemic hypersensitivity reactions were classified as anaphylaxis according to the National Institute of Allergy and Infectious Disease/Food Allergy and Anaphylaxis Network consensus criteria 18, and classified as severe according to the Brown grading system 19.

### Laboratory assays

A previously developed venom‐specific enzyme immunoassay was used to measure Russell's viper venom concentrations in serum samples 20, 21. Briefly, polyclonal IgG antibodies raised in rabbits against Russell's viper venom were bound to microplates and also conjugated with biotin as detecting antibodies for a sandwich enzyme immunoassay. The detecting agent was streptavidin–horseradish peroxidase. In cases where no pre‐antivenom sample was available or venom was not detected, a post‐antivenom sample was subjected to dissociation treatment so that any free venom could be measured with the enzyme immunoassay 22.

PT, INR, APTT, D‐dimer, FI (fibrinogen), FV, FVIII and FX were measured in citrated plasma samples. All assays were performed on a Sysmex CS‐2000i automated blood coagulation analyser (Sysmex Corporation, Chūō‐Ku, Kobe, Japan) with standard coagulometric or immunoturbimetric methods according to the manufacturer's protocols.

### Data analysis

The primary outcome was the proportion of patients with an INR of < 2 at 6 h post‐antivenom administration. The INR measured at the closest collection time before or after 6 h was used. Secondary outcomes included: (i) recurrence of coagulopathy defined as an increase in INR of > 2 in responding patients (INR of < 2 at 6 h); (ii) recurrence of venom antigenemia, defined as an increase in the detection of venom after an undetectable venom concentration 15; (iii) anaphylaxis resulting from antivenom or FFP; (iv) major hemorrhage as defined by the ISTH 23; (v) transfusion‐related lung injury (TRALI); and (vi) death prior to discharge. We also undertook an additional analysis that was not predefined, in which we only included patients who developed coagulopathy (INR of > 2, rather than a positive 20WBCT) and had an INR measured between 4 h and 9 h after antivenom administration.

In addition, the recovery of coagulopathy in the two groups was compared by the use of survival analysis, with the following events being used to measure recovery: time to fibrinogen of > 1 g L^−1^, INR of < 2, FV > 50%, and FX > 50%. The areas under the curve (AUCs) of D‐dimer were compared to determine whether FFP caused further consumption.

The sample size was based on a previous Australian study 24, in which ~ 25% of patients not given FFP recovered to an INR of < 2 after 6 h. This was confirmed in the recent controlled trial in Australia 5. We assumed that the process of recovery after antivenom administration and toxin neutralization was similar for different snakes. An absolute increase of 25% of patients recovering 6 h after antivenom administration was regarded as clinically significant and sufficient to warrant the use of FFP. In order to detect whether low‐dose antivenom and FFP increase the proportion of patients recovering at 6 h from 25% to 50%, with a significance level (alpha) of 5% and a power of 90%, a minimum of 85 patients were to be recruited to each arm (i.e. a total of 170 patients). We aimed to recruit 200 patients, to allow for missing outcome data.

There was no planned interim analysis, but a Data Monitoring Committee was established to review adverse effects. If > 10% of patients developed severe adverse effects attributed to FFP (anaphylaxis or TRALI), the study was to be stopped. Unfortunately, the rates of enrollment to the study decreased dramatically in the last year, owing to the sudden decrease in cases of Russell's viper envenoming in both hospitals. In this region of Sri Lanka, mechanical harvesting was introduced during the study, resulting in fewer Russell's viper bites. In addition, there was increased antivenom use at referral hospitals, reducing patient recruitment. The study was stopped after randomization of 141 patients of the 200, because grant funding had finished.

One author (K.M.), who remained blinded to treatment allocation, extracted and de‐identified the data from the trial database before classifying the primary and secondary outcomes. The chief investigator (G.I.), also blinded to treatment allocation, checked for missing or inconsistent data, and then unblinded treatment allocations supplied by the research team (F.M.).

### Statistical analysis

Medians and interquartile ranges (IQRs) were used for continuous variables, and 95% confidence intervals (CIs) were calculated for proportions. The primary outcome was analyzed by intention to treat with Fisher's exact test. A per‐protocol analysis and a non‐randomized analysis of treatment actually given were also undertaken. The latter compared patients who received high‐dose antivenom or low‐dose antivenom plus FFP within 6 h. Appropriate statistical tests were used for the secondary outcomes, based on whether the data were parametric or non‐parametric. Time to recovery of clotting (INR of < 2) and time to clotting factor recovery were analyzed with survival analysis and compared by use of the Gehan–Breslow–Wilcoxon test. Serial clotting time and clotting factor data were also plotted and compared visually. For factors for which there appeared to be a difference, a one‐phase association model was fitted to compare the half‐times of recovery. Statistical significance was taken as *P* < 0.05. All statistical analyses were performed with graphpad prism version 6.03 for Windows (GraphPad Software, San Diego, CA, USA; www.graphpad.com).

## Results

**Figure 1:**
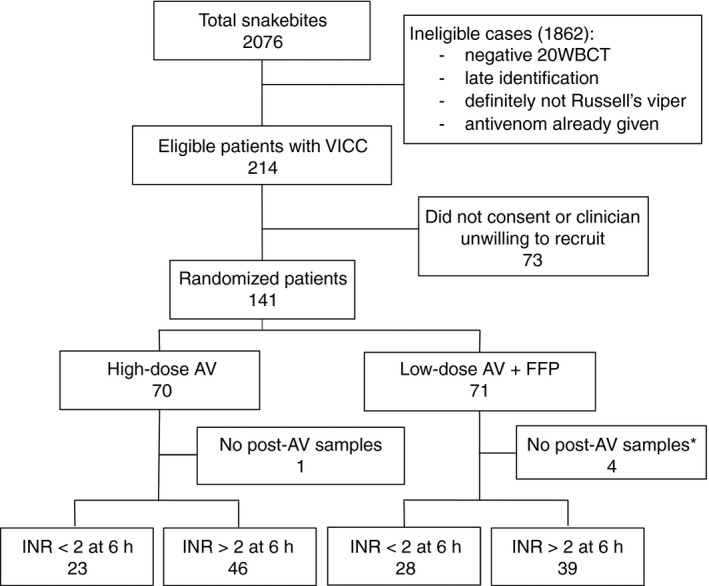
CONSORT diagram showing all snakebite patients presenting to both hospitals, the number of eligible patients, patients who did not consent, and patients for whom no outcome data were available because no blood was collected after antivenom (AV) administration. FFP, fresh frozen plasma; INR, International Normalized Ratio; VICC, venom‐induced consumption coagulopathy; 20WBCT, 20‐min whole blood clotting test.

From 214 eligible patients identified during the study period, 141 consented and were randomized; 71 to high‐dose antivenom alone, and 70 to low‐dose antivenom and FFP (Fig. 1). Five patients did not have blood collected after antivenom administration for the primary outcome, leaving 136 for the analysis. The patients allocated to each study arm had similar baseline characteristics, except that the patients allocated to high‐dose antivenom had higher venom concentrations and a statistically significantly shorter time to antivenom administration (3.1 h [IQR 2.3–4 h] versus 4.1 h [IQR 2.9–5.5 h]) (Table 1; Fig. S1). The use of 20WBCT to define coagulopathy resulted in 11 patients being recruited without coagulopathy being measured by a subsequent retrospective INR 25. In addition, six patients with hump‐nosed viper (*Hypnale* spp.) bites were inadvertently recruited (later identified by venom analysis), because this snake also causes coagulopathy and an abnormal 20WBCT 25, 26.

**Table 1:** Comparison of the demographic features and clinical effects for patients allocated to high‐dose antivenom and those allocated to low‐dose antivenom and fresh frozen plasma (FFP)

	High‐dose antivenom (*n* = 69)	%	Low‐dose antivenom + FFP (*n* = 67)	%
Sex (male)	54	78	52	78
Age (years), median (IQR)	38 (31–51)		45 (27–54)	
Snake type				
Russell's viper	63	91	60	90
Venom concentration (ng mL^−1^), median (IQR)	247 (24–692)		86 (26–312)	
Hospital				
Polonnaruwa	48	70	47	70
Kurunegala	21	30	20	30
Time to admission (h), median (IQR)	1.75 (1.1–2.5)		1.9 (1.2–3)	
Time to antivenom administration from bite, (h), median (IQR)	3.1 (2.3–4)		4.1 (2.9–5.5)*	

IQR, interquartile range. *One patient did not have a bite time; this was significantly different.

### Primary outcome

**Figure 2:**
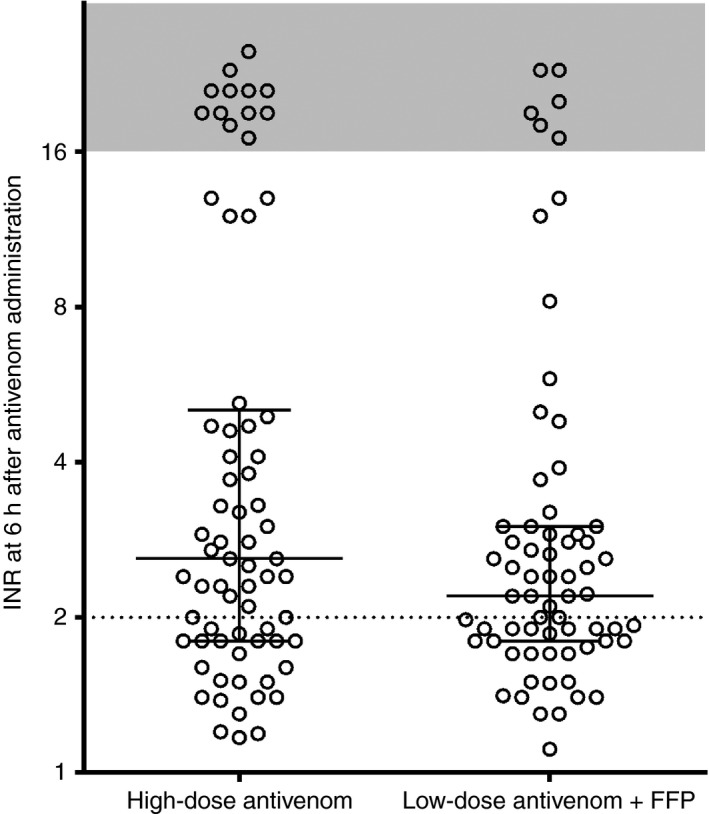
Scatter plot of the International Normalized Ratio (INR) measured at 6 h after antivenom administration, comparing those randomized to high‐dose antivenom with those randomized to low‐dose antivenom and fresh frozen plasma (FFP), for the intention to treat analysis. The shaded area represents an unrecordable INR (incoagulable blood).

**Figure 3:**
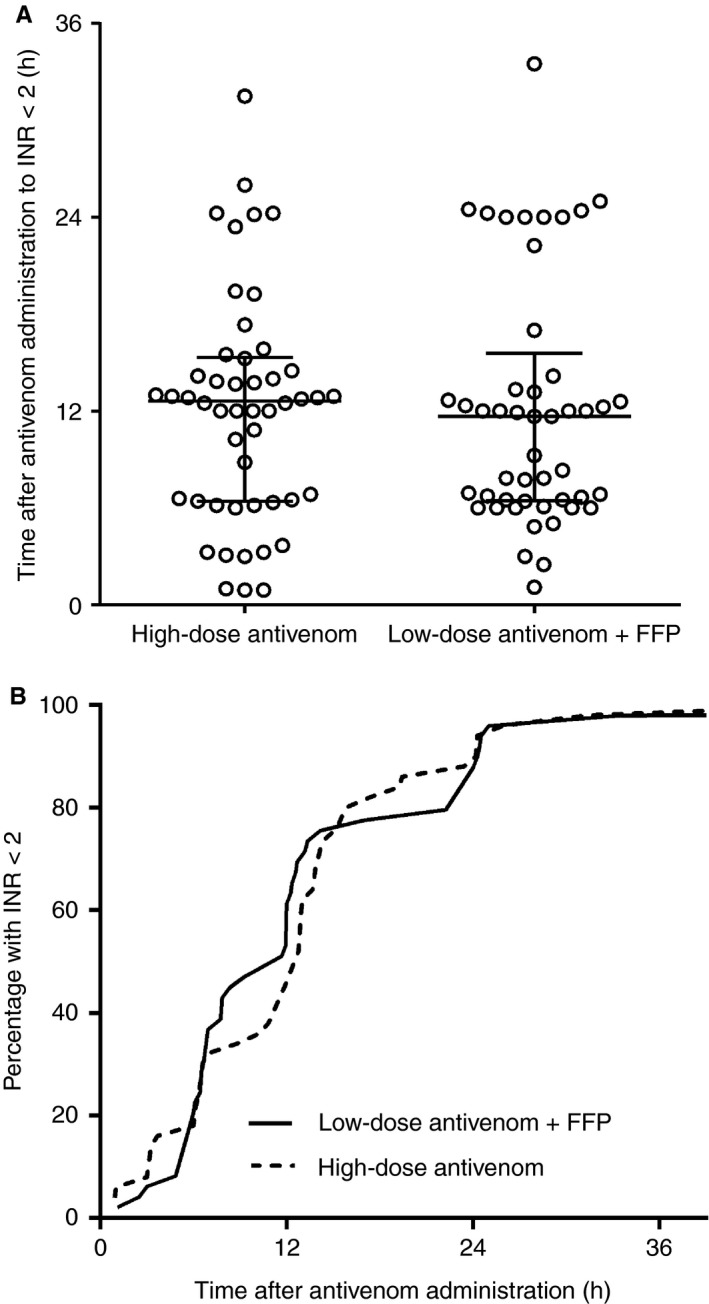
Scatter plot of the time until the International Normalized Ratio (INR) was < 2 (A) and a survival curve of the time to INR of < 2 (B), comparing patients randomized to high‐dose antivenom (thick line) with patients randomized to low‐dose antivenom and fresh frozen plasma (FFP) (dashed line), for the intention to treat analysis.

In an intention to treat analysis, 23 of 69 (33%) patients allocated to the high‐dose antivenom group had an INR of < 2 at 6 h after antivenom administration as compared with 28 of 67 (42%) allocated to the low‐dose antivenom plus FFP group (absolute difference of 8%; 95% CI − 8% to 25%) (Fig. 2). In a per‐protocol analysis, which was restricted to 119 patients receiving the allocated treatment, 22 of 65 (34%) given high‐dose antivenom had an INR of < 2 at 6 h after antivenom administration as compared with 25 of 52 (48%) receiving low‐dose antivenom and FFP (absolute difference of 14%; 95% CI − 4% to 32%). This per‐protocol analysis excluded four patients allocated to the high‐dose antivenom group, who were given FFP within 4 h, and 15 patients allocated to the low‐dose antivenom and FFP group, who were not given FFP (Fig. S2). The median time to an INR of < 2 after antivenom administration for patients given high‐dose antivenom was 12.9 h, as compared with 12 h (Gehan–Breslow–Wilcoxon test) for patients given low‐dose antivenom and FFP (Fig. 3). Thirty‐four patients of 136 either had an INR of > 2 for the last blood collection time point (right censored) or an INR that was never > 2 (excluded).

To assess the best possible effect of FFP, we included only the 118 patients who developed coagulopathy (INR of > 2, rather than a positive 20WBCT) and had an INR measured 4–9 h after antivenom administration. Of these 118, 17 of 72 (24%) given high‐dose antivenom had an INR of < 2 at 6 h, as compared with 21 of 46 (46%) who actually received low‐dose antivenom and FFP (absolute difference of 22%; 95% CI 5–39%) (Table 2).

**Table 2:** Additional primary outcome analyses compared with the Australian randomized controlled trial of fresh frozen plasma (FFP)

Primary outcome (INR of < 2 at 6 h)	*N*	High dose	%	*N*	Low dose + FFP	%	Difference (%)	(95% CI)
Intention to treat	69	23	33	67	28	42	8	− 8 to 25
Per protocol	65	22	34	52	25	48	14	− 4 to 32
Non‐randomized treatment with FFP excluding first INR of < 2* + INR at 3–9 h only†	72	17	24	46	21	46	22	5–39
Australian controlled trial of FFP‡	24	6	25	41	30	73	48	23–73

CI, confidence interval; INR, International Normalized Ratio. *Excluding all cases where the INR was < 2 on admission and remained at < 2. †For the primary outcome, the closest sample to 6 h after antivenom administration was used for analysis, including all samples; in this analysis, patients were excluded if the sample was taken earlier than 4 h or later than 9 h after antivenom administration. ‡In this study, patients were randomized to FFP or no FFP and the same antivenom dose.

### Secondary outcomes

Immediate systemic hypersensitivity reactions occurred in 50 of 140 patients in the study given antivenom with or without FFP, and 30 had severe anaphylaxis. There was no difference in reaction rates between high‐dose antivenom and low‐dose antivenom with FFP (Table 3). Administration of FFP was stopped after receipt of one unit in one patient, owing to the development of clinical features consistent with TRALI; this patient was then given further antivenom, and eventually died.

**Table 3:** Secondary outcomes for patients who received high‐dose antivenom compared with those who received low‐dose antivenom and fresh frozen plasma (FFP)

Outcome	*N*	High dose	%	*N*	Low dose + FFP	%	Difference (%)	(95% CI)
Systemic hypersensitivity reaction*	84	28	33	56	22	39	6	− 10 to 22
Severe anaphylaxis*	84	17	20	56	11	23	3	− 11 to 17
Recurrence								
Venom antigenemia	53	9	17	41	10	24	7%	− 9 to 24
Hypofibrinogenemia	74	1	1	52	1	2	–	–
Repeat antivenom dose (one or more)	69	27	39	67	18	27	15	− 2 to 32
Major hemorrhage	69	0		67	1		–	
Death	69	0		67	3		–	

CI, confidence interval. *One patient was not given antivenom.

A recurrence of the coagulopathy (INR of > 2) in patients with an INR of < 2 at 6 h occurred in two of 69 patients allocated to high‐dose antivenom, and in two of 67 in patients allocated to low‐dose antivenom and FFP. There were 94 patients in whom Russell's viper venom was detected in a pre‐antivenom sample. Venom recurrence after antivenom administration occurred in nine of 53 (17%) patients allocated to high‐dose antivenom, and in 10 of 41 (24%) patients allocated to low‐dose antivenom with FFP.

A repeat dose of antivenom was given to 27 of 69 (39%) patients given high‐dose antivenom, and to 18 of 67 (27%) patients given low‐dose antivenom. Four patients had a repeat dose within 6 h of antivenom administration, and all were in the low‐dose group. One patient was the patient who had FFP stopped because of becoming unwell. Two patients had repeat abnormal 20WBCTs after the first antivenom dose had been completed (2 h and 3 h after antivenom administration), and a further 10 vials were given. In a fourth patient, a repeat 20WBCT was performed during the infusion of the first antivenom dose, and was abnormal.

There were a total of three deaths, all in the low‐dose antivenom and FFP group, and all at one hospital. The first was a 56‐year‐old female who became acutely unwell immediately following the commencement of FFP and developed clinical features of TRALI, as well as acute kidney injury. She died on day 10. A 46‐year‐old male had a left parietal intracranial hemorrhage with extension into the ventricles and subarachnoid space with mass effect and uncus herniation. He died on day 3. The third was a 66‐year‐old female with diabetes and hypertension who presented with hematuria and acute renal failure, developed an ST elevation myocardial infarct on electrocardiogram, was positive for troponin I, and had a cardiac arrest and died 25 h post‐bite. There were no other intracranial hemorrhages or major hemorrhages.

### Clotting factor analysis

**Figure 4:**
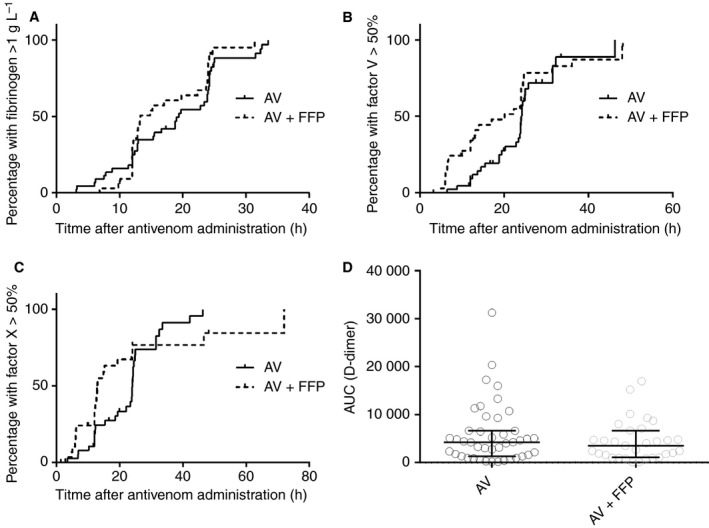
(A–C) Plots showing the proportion of patients with recovery of each factor versus time after antivenom (AV) administration, comparing those given high‐dose AV (black lines) with those given low‐dose AV plus fresh frozen plasma (FFP) (dashed lines), for recovery of fibrinogen (>1 g L^−1^ [A]), factor V (> 50% [B]), and factor X (> 50%; [C]). (D) Scatter plot of the area under the curve (AUC) of the D‐dimer measurements for each patient (g L^−1^ h), comparing patients given high‐dose AV with those given low‐dose AV with FFP.

There were 77 patients who had both detectable Russell's viper venom and sufficient timed samples for an analysis of all clotting factors; 44 received high‐dose antivenom and 33 received low‐dose antivenom and 4 U of FFP within 6 h. The median highest INR and APTT were > 12 (1.6 to > 12) and 180 s (28–180 s), respectively. The median lowest fibrinogen, FV, FX and FVIII levels and the median peak D‐dimer level were < 0.2 g L^−1^ (< 0.2 to 1.1 g L^−1^), 2.5% (2.5–45%), 25% (2.5–86%), 97% (2.5–292%), and 376 mg L^−1^ (4.8–909 mg L^−1^), respectively. There was no significant difference between groups. There was no significant difference in time to recovery of INR to 2 or fibrinogen to 1 g L^−1^ (Fig. 4A). There was statistically significantly more rapid recovery of FV (Gehan–Breslow–Wilcoxon test; Fig. 4B) and FX (Fig. 4C) in the low‐dose antivenom group within 24 h. There was no significant difference in the D‐dimer AUC between groups (median 4208 g L^−1^ h versus 3469 g L^−1^ h; Fig. 4D). Visual inspection of plots of INR, fibrinogen, FV, FVIII, FX and D‐dimer versus time, comparing the two groups, showed little difference in recovery of factors except for FV (Fig. S3). Timed fibrinogen, FV and FX data were then globally fitted to a one‐phase association equation for those receiving FFP versus those not receiving FFP (Fig. S4). The half‐time of recovery of FV for patients receiving FFP was 13.2 h (95% CI 8.3–38 h), which was shorter than that for those not receiving FFP, i.e. 24.2 h (95% CI 13–200 h).

## Discussion

The study has two important findings. The first is that the lower dose of 10 vials of antivenom appeared to be as effective as the higher dose. The second, more interesting, finding is that, although FFP appeared to more rapidly improve the coagulopathy in some patients, there was no significant overall benefit. Our pragmatic study of clinical effectiveness had the same difficulties in identifying patients who would benefit from and received FFP, as would be experienced in non‐trial settings; only 77% of those allocated to the FFP arm actually received it. Thus, the study demonstrates the lack of general clinical benefit of attempted routine use of a set dose of FFP in this resource‐poor setting. This is in contrast to what would be expected to happen (say) with FFP given to an individual patient with a clearly documented high INR and life‐threatening bleeding after VICC.

There was a significant but small difference in median time to antivenom of 1 h, with administration of antivenom being delayed in the low‐dose antivenom group. This may have been because organizing a cross‐match and planning administration of FFP slowed the process of giving antivenom. This increase from a median of 3–4 h post‐bite is potentially clinically significant. Some studies have shown that the benefit of antivenom is measured in hours, particularly within the first 6 h post‐bite 27, 28.

Administration of FFP can only benefit patients who have developed coagulopathy. Eleven patients who never developed coagulopathy were included because they had a positive 20WBCT. We have previously outlined the poor sensitivity of the 20WBCT for detecting VICC, but found a poor specificity in patients recruited to this study 25. If a laboratory INR had been used to determine whether patients had VICC, these patients would have been excluded. Predictably, we observed a greater effect of FFP when these patients were excluded from analyses (Table 2).

The graphs of the time to recovery of fibrinogen, FV and FX suggest that, in the FFP group, there is more rapid recovery of these factors in the first 24 h than in the high‐dose antivenom group (Fig. 4); this is only significant for FV and FX. However, on further analysis of serial factor concentrations, only FV had a shorter half‐time of recovery (13.6 h versus 24.2 h; Figs S3 and S4). After 24 h, the recovery appeared to be similar in both groups, indicating that FFP provides a transiently more rapid improvement for some factors. Even when patients unlikely to benefit from FFP were excluded, and acknowledging that this was not a prespecified outcome, the magnitude of the absolute difference in this study was still only half that seen in the Australian study (Table 2). Despite the Australian study of FFP in snakebite showing a highly significant improvement in coagulopathy, it did not lead to earlier discharge or clinical benefit 5.

There were three deaths in the low‐dose antivenom group, but only one appeared to be related to FFP treatment, with TRALI developing. However, the diagnosis of TRALI could not be confirmed, the reaction occurred immediately after administration of 1 U of FFP, and the patient developed acute kidney injury related to envenoming. The patient immediately received further doses of antivenom. The other two patients developed severe complications of envenoming, and also received further antivenom doses.

A potential limitation of the study was that it was underpowered, with only 136 patients being included in the intention to treat analysis, rather than the 170 patients specified by the sample size calculation. It is possible that an additional 30 patients may have produced a positive trial with a very small effect size, but this would have still not been clinically beneficial.

Another major limitation of the study was the number of patients randomly allocated to the low‐dose antivenom and FFP group who did not receive FFP. In the majority of cases, this was attributable to the inability to cross‐match the patient (Fig. S2), although, in some cases, it was attributable to changing shifts or new doctors refusing to administer the treatment after inclusion in the study. This reduced the effective power of the study, and is one potential reason for the negative intention to treat outcome, despite a favorable trend in patients actually given FFP. Almost one‐third of patients received further antivenom, but this was given 6 h or more after the initial dose in all but four cases.

Patients allocated to low‐dose antivenom and FFP received antivenom 1 h later than the high‐dose group. This may have affected the outcome, biasing a successful outcome to the high‐dose group. However, a delay in antivenom administration of 1 h is unlikely to have a major effect on FFP administration, and all patients allocated to FFP and who were given FFP received it within 6 h. This study adds further to the evidence base on FFP in VICC, which is a major complication of many snakebites worldwide. In contrast to the Australian study 5, we found that FFP was only beneficial in a subgroup of patients who were not easily identified in a resource‐poor setting where 20WBCT is relied on for diagnosis. In addition, the use of FFP appeared to delay antivenom adminstration, and it was sometimes impractical to administer. Although we were not able to demonstrate that FFP improved rates of recovery from VICC in this setting, we saw no evidence that it worsened or prolonged coagulopathy. This is reassuring for its potential role in treating hemorrhagic complications, and also encourages further studies with other snake species, such as *Echis* spp. (saw‐scaled and carpet vipers) 8, *Crotalus* spp. and *Bothrops* spp. (vipers from the Americas) 29, and *Calloselasma rhodostoma* (Malaysian pit viper)^30^. As many of these snakes occur in resource‐poor parts of the world, routine FFP administration is likely to be of limited clinical benefit, so the focus should remain on early administration of antivenoms.

## Addendum

G. K. Isbister, A. H. Dawson, D. G. Lalloo, H. J. de Silva, and N. A. Buckley designed the study. F. Mohamed randomized patients. F. Mohamed, G. K. Isbister, I. Gawarammana, and S. Jayamanne recruited and/or supervised the recruitment of patients and data collection. G. K. Isbister, K. Maduwage, and F. Mohamed performed the data audit. F. E. Scorgie, L. F. Lincz, and K. Maduwage undertook all assays. G. K. Isbister and N. A. Buckley analyzed the data. G. K. Isbister wrote the first draft and takes responsibility for the study. All authors contributed to the final manuscript.

## Disclosure of Conflict of Interests

The authors state that they have no conflict of interest.

## Supporting information


**Fig. S1.** Scatter plot of the time to antivenom comparing patients given high‐dose antivenom to those given low‐dose antivenom with FFP.
**Fig. S2.** Flow chart showing all allocated patients, the number included in the intention to treat analysis and then the patients not given their allocated treatment (excluded in the per‐protocol analysis) and who then changed arms for the non‐randomized analysis because either FFP was administered when it should not have been, or was not administered when it should have been.
**Fig. S3.** Plots of International Normalized Ratio (INR; A, B), fibrinogen (C, D), factor V (E, F), factor VIII (G, H) and factor X (I, J) versus time comparing those given high‐dose antivenom (left‐sided graphs) versus those receiving low‐dose antivenom and FFP (right‐sided graphs). Note: factor VIII is initially high because of the artificial effect that Russell's viper venom has on the factor VIII assay (see Isbister *et al*.) 13
**Fig. S4.** Predicted time course of factor V (red), factor X (black) and fibrinogen (blue), comparing FFP (thick line) to no FFP (dashed line), when the observed data were fitted to a one‐phase association model demonstrating the early increase in factor V in the FFP group.
