# Supplementary material for: A randomized controlled trial of fresh frozen plasma for coagulopathy in Russell's viper (Daboia russelii) envenoming
Source: J Thromb Haemost. 2017 Feb 16;15(4):645–54. doi: 10.1111/jth.13628 (PMC5408386; doi:10.1111/jth.13628)
Supplement: Supplementary file 1 — Fig. S1. Scatter plot of the time to antivenom comparing patients given high‐dose antivenom to those given low‐dose antivenom with FFP. Fig. S2. Flow chart showing all allocated patients, the number included in the intention to treat analysis and then the patients not given their allocated treatment (excluded in the per‐protocol analysis) and who then changed arms for the non‐randomized analysis because either FFP was administered when it should not have been, or was not administered when it should have been. Fig. S3. Plots of International Normalized Ratio (INR; A, B), fibrinogen (C, D), factor V (E, F), factor VIII (G, H) and factor X (I, J) versus time comparing those given high‐dose antivenom (left‐sided graphs) versus those receiving low‐dose antivenom and FFP (right‐sided graphs). Note: factor VIII is initially high because of the artificial effect that Russell's viper venom has on the factor VIII assay (see Isbister et al.) 13 Fig. S4. Predicted time course of factor V (red), factor X (black) and fibrinogen (blue), comparing FFP (thick line) to no FFP (dashed line), when the observed data were fitted to a one‐phase association model demonstrating the early increase in factor V in the FFP group. [file JTH-15-645-s001.docx]

***Supplementary Figure 1:*** Scatter plot of the time to antivenom comparing patients given high-dose antivenom to those given low-dose antivenom with FFP.

***Supplementary Figure 2*:** Flow chart showing all allocated patients, the number included in the intention to treat analysis and then the patients not given their allocated treatment (excluded in the per-protocol analysis) and who then changed arms for the non-randomized analysis because either FFP was administered when it should not have been, or was not administered when it should have been.

***Supplementary Figure 3*:** Plots of international normalized ratio (INR; Panels A and B), fibrinogen (Panels C and D), factor V (Panels E and F), factor VIII (Panels G and H) and factor X (Panels I and J) versus time comparing those given high-dose antivenom (left-sided graphs) versus those receiving low-dose antivenom and FFP (right-sided graphs). Note. Factor VIII is initially high because of the artificial effect that Russell’s viper venom has on the factor VIII assay (see Isbister et al.)^8^

***Supplementary Figure 4:*** Predicted time course of factor V (red), factor X (black) and fibrinogen (blue), comparing FFP (thick line) to no FFP (dashed line), when the observed data was fitted to a one-phase association model demonstrating the early increase in factor V in the FFP group.

**Supp Figure 1**





**Supp Figure 2**

**
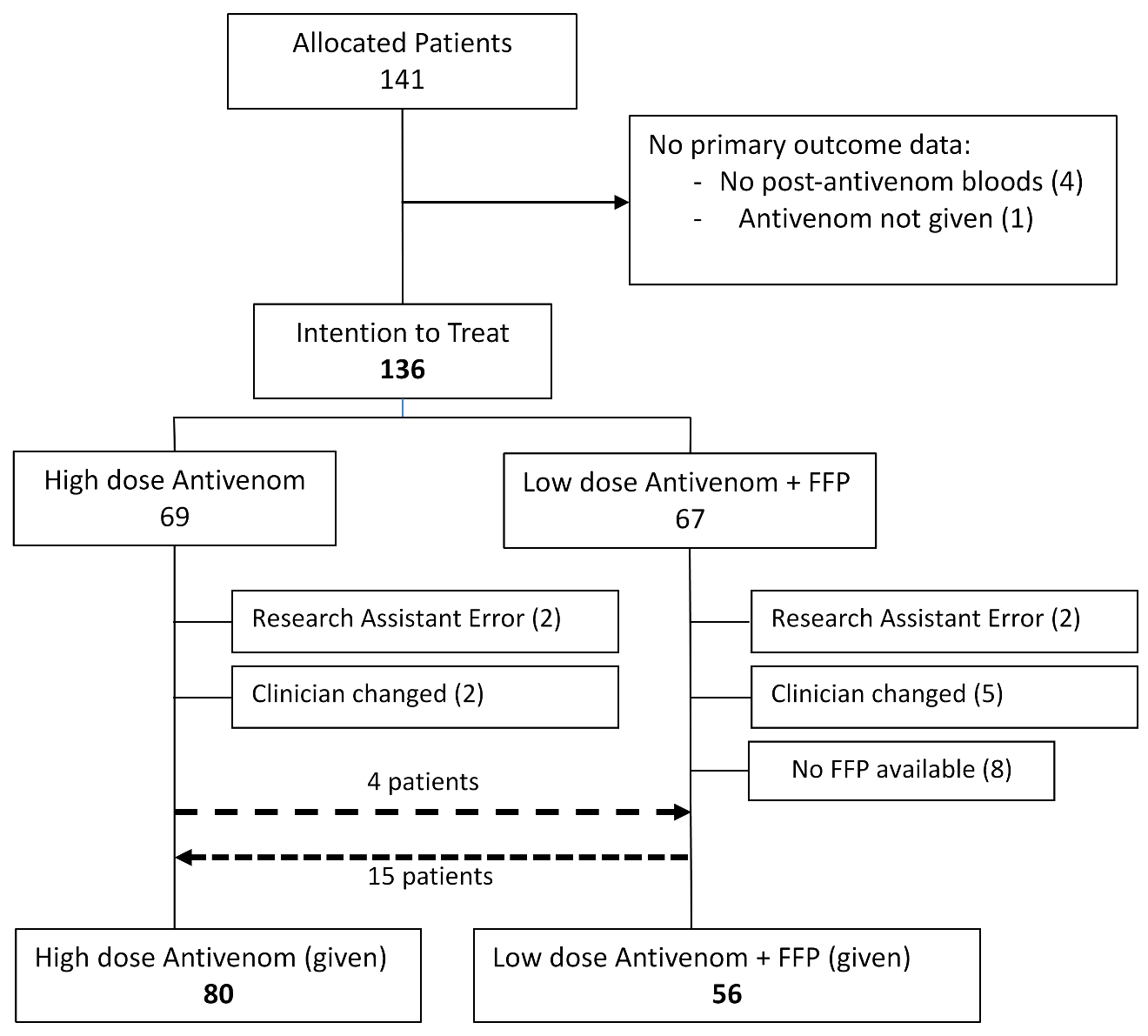
**

**Supp Figure 3**





**Supp Figure 4**
